# In vitro and in vivo studies reveal α-Mangostin, a xanthonoid from *Garcinia mangostana*, as a promising natural antiviral compound against chikungunya virus

**DOI:** 10.1186/s12985-021-01517-z

**Published:** 2021-02-28

**Authors:** Poonam Patil, Megha Agrawal, Shahdab Almelkar, Manish Kumar Jeengar, Ashwini More, Kalichamy Alagarasu, Naveen V. Kumar, Prathama S. Mainkar, Deepti Parashar, Sarah Cherian

**Affiliations:** 1 ICMR-National Institute of Virology, 20-A, Dr. Ambedkar Road, Pune, Maharashtra 411001 India; 2 CSIR-Indian Institute of Chemical Technology [CSIR-IICT, Hyderabad, 500 007 India

**Keywords:** Α-Mangostin, Treatment, Chikungunya, Antiviral therapy, Xanthonoids

## Abstract

**Background:**

Chikungunya virus (CHIKV), a serious health problem in several tropical countries, is the causative agent of chikungunya fever. Approved antiviral therapies or vaccines for the treatment or prevention of CHIKV infections are not available. As diverse natural phenolic compounds have been shown to possess antiviral activities, we explored the antiviral activity of α-Mangostin, a xanthanoid, against CHIKV infection.

**Methods:**

The in vitro prophylactic and therapeutic effects of α-Mangostin on CHIKV replication in Vero E6 cells were investigated by administering it under pre, post and cotreatment conditions. The antiviral activity was determined by foci forming unit assay, quantitative RT-PCR and cell-based immune-fluorescence assay. The molecular mechanism of inhibitory action was further proposed using in silico molecular docking studies.

**Results:**

In vitro studies revealed that 8 µM α-Mangostin completely inhibited CHIKV infectivity under the cotreatment condition. CHIKV replication was also inhibited in virus-infected mice. This is the first in vivo study which clearly showed that α-Mangostin is effective in vivo by significantly reducing virus replication in serum and muscles. Molecular docking indicated that α-Mangostin can efficiently interact with the E2–E1 heterodimeric glycoprotein and the ADP-ribose binding cavity of the nsP3 macrodomain.

**Conclusions:**

The findings suggest that α-Mangostin can inhibit CHIKV infection and replication through possible interaction with multiple CHIKV target proteins and might act as a prophylactic/therapeutic agent against CHIKV.

**Supplementary Information:**

The online version contains supplementary material available at 10.1186/s12985-021-01517-z.

## Background

Chikungunya fever (CHIKF), caused by chikungunya virus (CHIKV) is frequently associated with a high prevalence of chronic arthralgia. After the acute phase, polyarthritis can be recurrent and may persist for several years after infection [1]. This is a serious public health concern in tropical countries throughout Africa and Asia. More recently, CHIKV is emerging in temperate areas such as Europe and the America [2]. Except for prescribing symptomatic treatments and treatment with nonsteroidal anti-inflammatory drugs, there are no specific drugs or vaccines available for treating or preventing CHIKV infections [3, 4].

Similar to other members of the alphavirus genus, the CHIKV starts its life cycle by entering the target host cells via receptor-mediated endocytosis [5]. The viral envelope proteins (E1 and E2) play a major role by binding and fusion with the infected cell surfaces [6]. The non structural proteins (nsP1-4) along with performing a variety of intracellular functions are known to be the primary mediators of viral replication. The nsP1 of alphaviruses show cap methyl- and guanylyltransferase activities and nsP2 has protease, NTPase, RNA triphosphatase, and RNA helicase activities [7–9]. The nsP3 includes an amino-terminal macro domain and a central alphavirus-specific zinc-binding domain (ZBD). The nsP4 is the viral RNA dependent RNA polymerase (RdRp) and is directly responsible for the replication of viral RNA [10–12].

Natural polyphenolic compounds like flavonoids and xanthonoids possess wide ranging bioactivities such as anti-inflammatory, anti-oxidative, anti-bacterial, anti-fungal as well as antiviral activities [13–16]. Among the diverse flavonoids, Baicalein, fisetin, quercetagetin, silymarin, curcumin, nobiletin displayed potent inhibition of CHIKV infection [17–19]. Among xanthonoids, the bioactivity of specific compounds of *Garcinia mangostana* pericarp (GMP) extracts, including α-Mangostin, β-Mangostin and γ-Mangostin have been better elucidated [20]. *Garcinia mangostana* (Mangosteen) Linn belongs to the Clusiaceae family and is cultivated mainly in Malaysia, Thailand, Indonesia, Philippines, Sri Lanka and India [21]. α-Mangostin is known for its medicinal use in a variety of clinical problems such as diarrhea, dysentery, diabetes, convulsion, inflammation, ulcers, wound healing as well as cancer [21, 22]. The antiviral activity of α-Mangostin was first reported in 1996 by Chen et al., where the ethanolic extract of the fruit peel from the plant showed potent inhibitory effect against HIV-1 protease. In the recent past, antiviral effects of α-Mangostin have also been reported against HCV virus and dengue virus [22–26]. There are no reports on the potential antiviral activity of α-Mangostin against CHIKV. Hence, in this study, we explored the in vitro and in vivo antiviral activity of α-Mangostin against CHIKV infection and further investigated the mechanism of possible action using in silico methods.

## Materials and methods

### Cells, virus and chemicals

Vero E6 cells and the CHIKV strain of African genotype (Strain No. 061573; Andhra Pradesh 2006; GenBank Accession Number EF027134) were used. Modified Eagle’s medium was used to maintain the Vero E6 cell line supplemented with 5% Fetal bovine serum (FBS, Gibco, Technologies, NY,US) and anti mycotic antibiotic solution (Sigma Aldrich, US). α-Mangostin, a natural compound used for this study was obtained from the National MolBank compound repository of CSIR-IICT, Hyderabad. The purity of the compound was determined to be 83% by HPLC.

### Mice

For induction of chikungunya disease, female C57BL/6 mice (n = 9) of 4–5 weeks age were obtained from the institutional animal breeding facility. Animals were randomly selected, marked to permit individual identification, and kept in their cages for at least one week prior to the start of the dosing to allow for acclimatisation to laboratory conditions. All animal experiments were done in a biosafety level-2 animal facility at the ICMR-NIV and the study was approved by the Institutional Animal Ethics Committee (IAEC) and Institutional Bio-safety committee [27]. Animal housing and care protocols were followed as per the Committee for the Purpose of Control and Supervision of Experiments on Animals (CPCSEA) guidelines.

### Assessment of the cytotoxicity of α-Mangostin on Vero E6 cells

To evaluate the cytotoxic effect of α-Mangostin on Vero E6 cells, MTT assay was used as reported previously [28].

### Assessment of the antiviral activity of α-Mangostin in cells

For studying the effect of pretreatment of cells with α-Mangostin, the cells were pretreated with different concentrations of the α-Mangostin for 4 h followed by infection, while in case of cotreatment, the virus was mixed with different concentrations of α-Mangostin and immediately used for infecting the cells for 1 h. From the virus stock with the known titre of FFU/ml, a multiplicity of infection (M.O.I) of 0.01 was used in all the antiviral assays. After infection, the culture supernatant was replaced and incubated for 24 h. To study the effect of α-Mangostin post infection, the cells were first infected and after 1 h incubation, the virus was removed and 2 washes of sterile PBS was given to cells to remove unbound virus particles. After washing, various concentrations of α-Mangostin were added to wells according to standard protocol in triplicates and incubated for 24 h. After 24 h, the plate was freezed for further assays. The vehicle control (VC) contained DMSO diluted with the working media in the same ratio as in the highest concentration treatment group (8 µM). In all conditions, after the incubation, the culture supernatant was assessed for viral load in terms of viral genomic RNA levels by real time RT-PCR and infectious virus particles by focus forming unit (FFU) assay. The magnitute of the change in viral load was measured on a logarithmic scale and log reduction to percentage reduction was calculated as reported previously [29]. For assessing the effect of α-Mangostin on infectivity, immunoflourescence assay (IFA) was used. All the experiments were performed in triplicates.

### FFU assay

FFU assay was performed as reported earlier with modifications [30]. The primary in house developed mouse anti-chikungunya antibody (1:300) and goat anti mouse IgG HRP conjugate (Sigma-Aldrich St. Louis, MO, USA) (1:1000) as secondary antibody was used.

### Immunofluorescence assay

Approximately equal number of Vero E6 cells (2 × 10^5^) were seeded in a 24 well plate (Tissue Culture Test Plate 24, TPP, Switzerland) with a coverslip placed in each well. The cells were allowed to form a confluent monolayer and were infected with CHIKV and incubated for 12 h. After incubation, the immunofluorescence assay (IFA) was performed as reported in a previous study [28]. An in house developed monoclonal antibodies against the capsid protein of CHIKV was used was used as the primary antibody [31].

For quantification of the proportion of infected cells, the total cells as well as the infected cells were counted in 10 randomly selected fields per well and percentage of infection was calculated based on the ratio of infected cells in a field to total number of cells in that field.

### Quantitative reverse transcription polymerase chain reaction (qRT-PCR)

qRT-PCR measurements studies were performed for quantitating viral RNA copy number. Briefly, viral RNA was extracted from Vero E6 cell culture supernatants, serum and muscle tissues using RNA extraction kits (Qiagen, Hilden, Germany). One step real time RT-PCR was performed as reported previously [28]. The primers and the probe targeted the E3 gene and the sequences have been reported earlier [32]. The RNA copy number of the sample was calculated using a standard curve generated employing *in-vitro* transcribed RNA standards.

### Assessment of antiviral activity of α-Mangostin in mice

Female C57BL/6 mice of 4 week age were used to investigate the anti-CHIKV activities of α-Mangostin*.* Mice were infected with 1 × 10^6^ pfu of CHIKV by intramuscular (i.m.) route and viral RNA copies were assessed in serum and muscles on the 3rd, 5th, and 7th day post infection (dpi). α-Mangostin was diluted in normal saline with 0.5% sodium salt of carboxy methyl cellulose (CMC) as the vehicle, and two doses, 20 mg/kg and 40 mg/kg, were administered at 1st, 2nd and 3rd dpi via the intraperitoneal (ip) route. As per the recommended volume (10 ml/kg), of a vehicle for the IP route, the mice (body weight ~ 25 gm) were administered 250 µl (1.25 mg) of CMC solution. Mice from the VC group received the same amount of the CMC solution without mangostin to observe any possible effect of the vehicle. Sodium carboxy methyl cellulose was used since it is a good dispersing agent, viscosity modifier and emulsifier [33]. At the end of the study, mice were euthanized followed by cervical dislocation. Serum and muscle tissues were collected for RT-PCR and histopathology analysis at 3rd, 5th, 7th dpi.

### Histopathology

Histopathological evaluation was performed on the muscle tissues of the hind limb from the Normal (saline injected), CHIKV infected (3rd, 5th and 7th dpi), and CHIKV infected mice treated with different doses of α-Mangostin (20 mg/kg and 40 mg/kg). Hind limb tissues, excluding the femur bone, were fixed in 10% formaldehyde as per the standard procedure and processed for histopathology. Paraffin sections of 5 µm thickness were prepared, stained with haematoxylin and eosin.

### Compound, molecular modeling and docking studies with viral target proteins

The 2-dimensional structure of α-Mangostin was generated using ChemDraw® Professional 16.0.1.4 software. All available crystal structures of the CHIKV target proteins in the Protein Data Bank (PDB) including the CHIKV envelope glycoprotein complex in its mature form (3N42.PDB), CHIKV nsP3 macrodomain bound with ADP-ribose (3GPO.PDB) and the CHIKV peptidase C9 domain of nsP2 (3TRK.PDB) were retrieved. For modeling the structures of the nsp1 v-methyltranseferase domain, nsp2 helicase domain and nsp4 RdRp domain, the protein sequences of the CHIKV targets were obtained from the NCBI GenBank (accession nos. ARB19731). As no homologous protein structure templates were available in PDB for these CHIKV proteins, all structure modelling studies were carried out using the on-line Local Meta-Threading-Server LOMETS, for protein structure prediction (https://zhanglab.ccmb.med.umich.edu/LOMETS/). The modeled protein structures were further validated using PROCHECK (http://servicesn.mbi.ucla.edu/PROCHECK) and ProQ (https://proq.bioinfo.se/cgi-bin/ProQ/ProQ.cgi).

The ligand–protein docking interactions of all the CHIKV target crystal structures were simulated using AutoDock Vina [34]. All the target structures were preprocessed and minimized by adding polar hydrogens and gasteiger charges using Autodocktools (ADT). The grid box parameters were set in such a way that the search is performed over the entire protein surface. Default values were used for all the other docking parameters. The ligand for the docking studies was also preprocessed by AutodockTool (ADT). In case of the nsP3 macrodomain, the coordinates of ADP-ribose, the natural substrate, were deleted during the docking study. The binding site predictions prior to docking studies, the interaction analysis and molecular visualization of docked complexes were performed using BIOVIA Discovery Studio 2017 R2 software package.

### Statistical analysis

All data are represented as mean ± SD and statistical analysis was performed using GraphPad Prism 5.0 software, utilizing one-way ANOVA and Dunnett’s multiple comparison test. *p* values < 0.05, were considered significant. For each time point, three replicates were used and all experiments were performed three times.

## Results

### Effect of α-Mangostin treatment on proliferation of Vero E6 cells (MTT assay)

The effect of α-Mangostin on Vero E6 cells viability was investigated using the MTT assay. The MTT assay revealed that ≤ 8 μM concentrations of α-Mangostin had no effect on cell viability (viability ≥ 90%) (CC50 14 µM, Additional file 1: Fig. S1). Hence, concentrations from 1 to 8 μM of α-Mangostin were used in further experiments.

### Effect of α-Mangostin on CHIKV replication

**Fig. 1:**
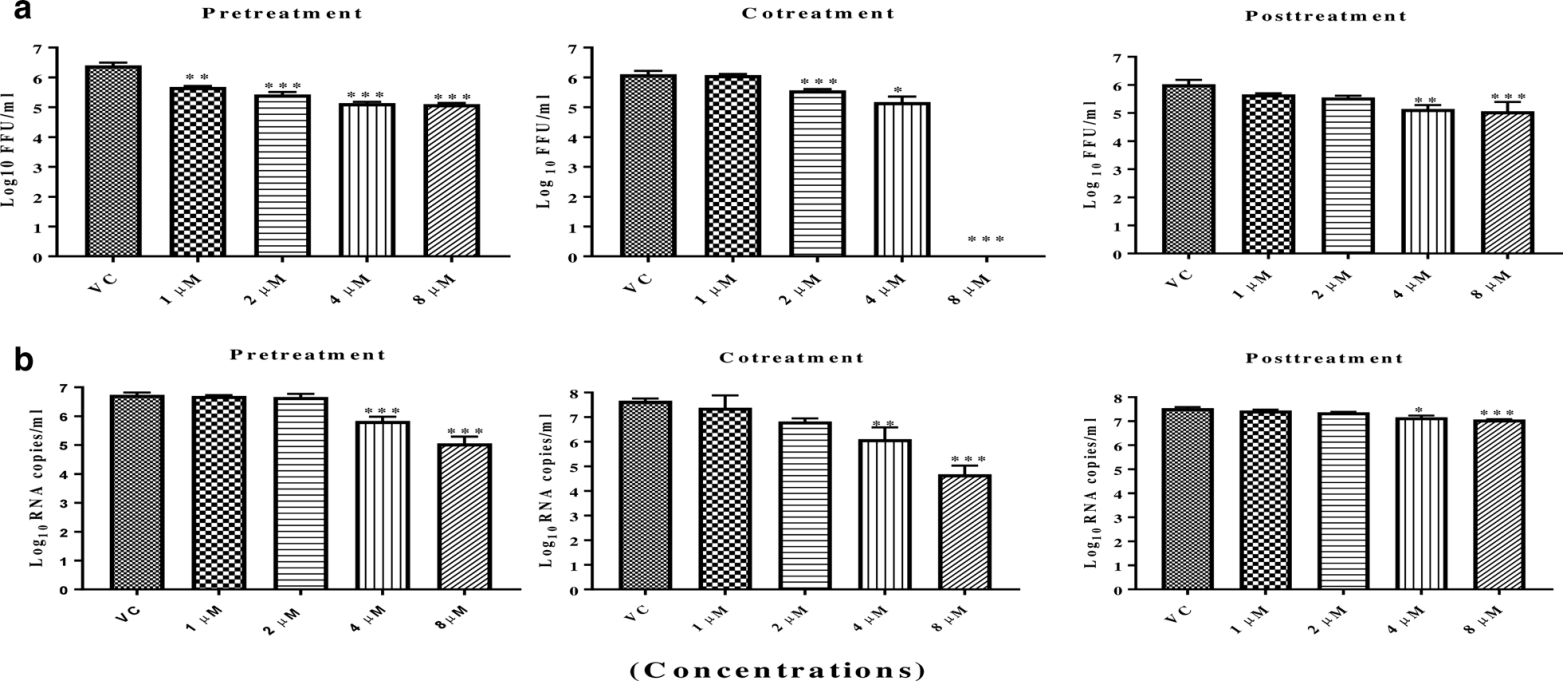
Effect of α-Mangostin on CHIKV as assessed by focus forming unit assay (**a**) and qRT-PCR assay (**b**) under pretreatment, cotreatment and posttreatment conditions at 24 h. Vero E6 cells were pre, co and posttreated with different concentrations (1, 2, 4 and 8 µM) of α-Mangostin. After 24 h incubation, the plates were freezed and the culture filtrates were used for the different assays. For the FFU assay, mouse anti-chikungunya antibody and goat anti mouse IgG HRP conjugate as primary and secondary antibodies were used. For qRT-PCR total RNA was isolated and CHIKV RNA was detected by measuring E3 RNA copies by real-time RT-PCR. The titres are presented as log_10_ titres ***p < 0.001; *p < 0.05. All the values are expressed as mean ± SD. The experiments were performed in triplicates in three independent trials. ***p < 0.001; **p < 0.01 and *p < 0.05 vs. vehicle control

Vero E6 cells were infected with CHIKV and treated with different concentrations (1 μM, 2 μM, 4 μM and 8 μM) of the α-Mangostin for before, after and simultaneous infection and the FFU assay and Quantitative RT-PCR was performed at 24 h. In FFU assay, a dose dependent reduction in viral titers with respect to CHIKV was noted for pretreatment, co-treatment and post-treatment conditions (Fig. 1a). The pretreatment of cells with 8 µM α-Mangostin 4 h prior to CHIKV infection reduced the CHIKV titre from 6.40 to 5.10 mean log_10_ FFU/ml (95% reduction in FFU titre) (*p* < 0.001). When the virus was mixed with α-Mangostin and used for infection (cotreatment) 100% reduction of the virus titer was noted at 8 µM concentration (Fig. 1a). Treatment of the cells 4 h post infection resulted in foci reduction from 6.02 to 5.06 mean log_10_ FFU/ml (89% reduction in FFU titre) (*p* < 0.001).

Quantitative RT-PCR showed a significant 1 log and 3 log titer (*p* < 0.001) decrease in copy number of CHIKV RNA for pre and cotreatment conditions using 8 μM α-Mangostin respectively compared to the untreated cells while a < 1 log reduction in the CHIKV RNA copy number was observed under post-treatment conditions (*p* < 0.05 for 4 µM) and (*p* < 0.001 for 8 μM) (Fig. 1b).

**Fig. 2:**
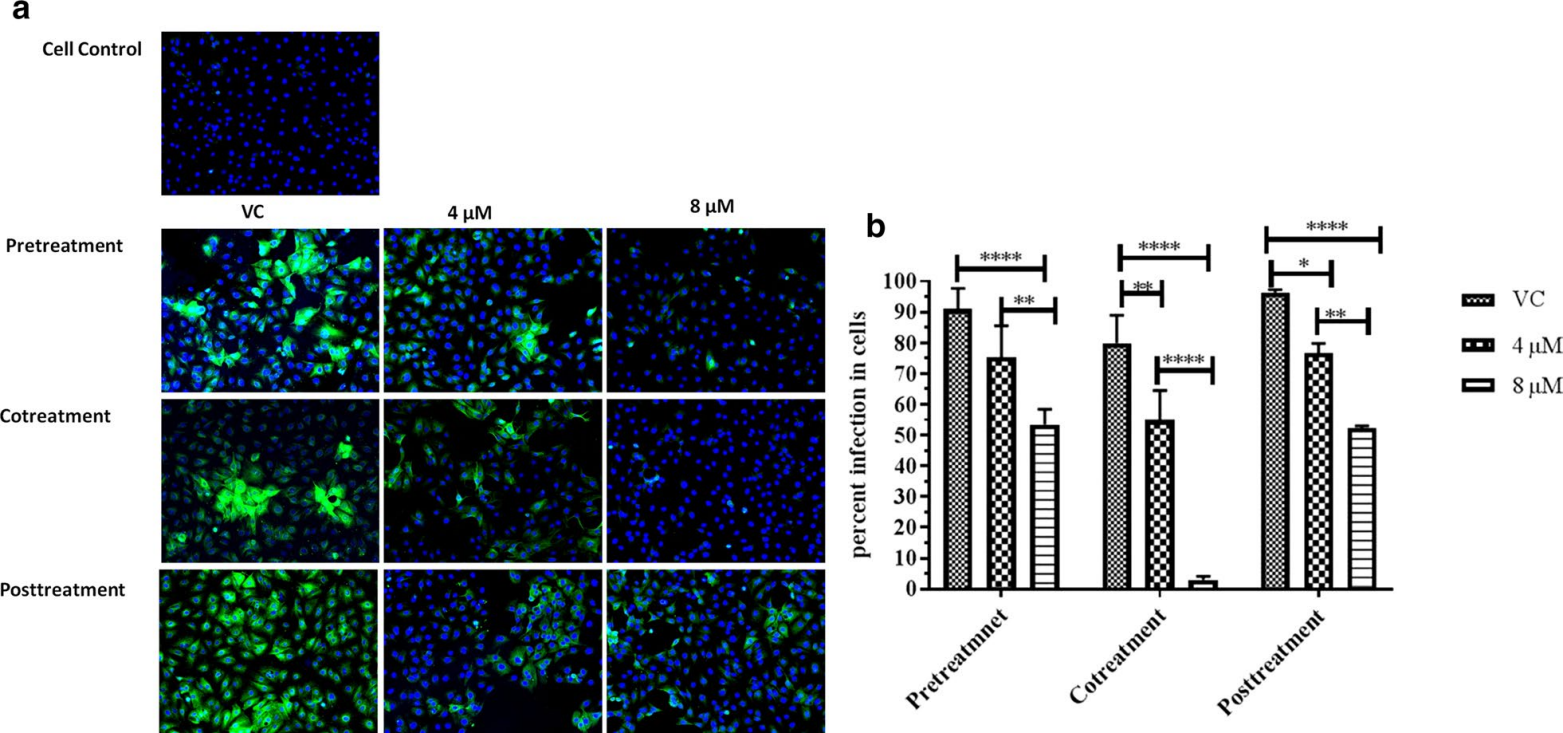
Immunoflouresce assay **a** immunoflourescent images of CHIKV infected Vero E6 cells under pre-treatment, posttreatment and cotreatment conditions. Virus infected cells appear green in colour; **b** percentage of infected Vero E6 cells in cultures infected with virus pretreated, cotreated and posttreated with different concentrations of α-Mangostin. Cells were counted in three different fields to obtain the percent infected cells. All the values are expressed as mean ± SD. The experiments were performed in triplicates in three independent trials. ***p < 0.001; **p < 0.01 and *p < 0.05 vs. vehicle control

IFA results show that there was a strong inhibition of expression of viral antigens and dose dependent reduction in the percentage of infected cells pretreated with α-Mangostin (Fig. 2a, b). Similar reduction in infection was observed in cells infected with virus cotreated with α-Mangostin compared to the VC. Though, the reduction in the number of infected cells were observed in cells treated with α-Mangostin after infection compared to the VC, the effect was not as prominent as that observed in pretreatment and cotreatment conditions.

### Inhibition of the CHIKV replication in C57BL/6 mice treated with α-Mangostin

**Fig. 3:**
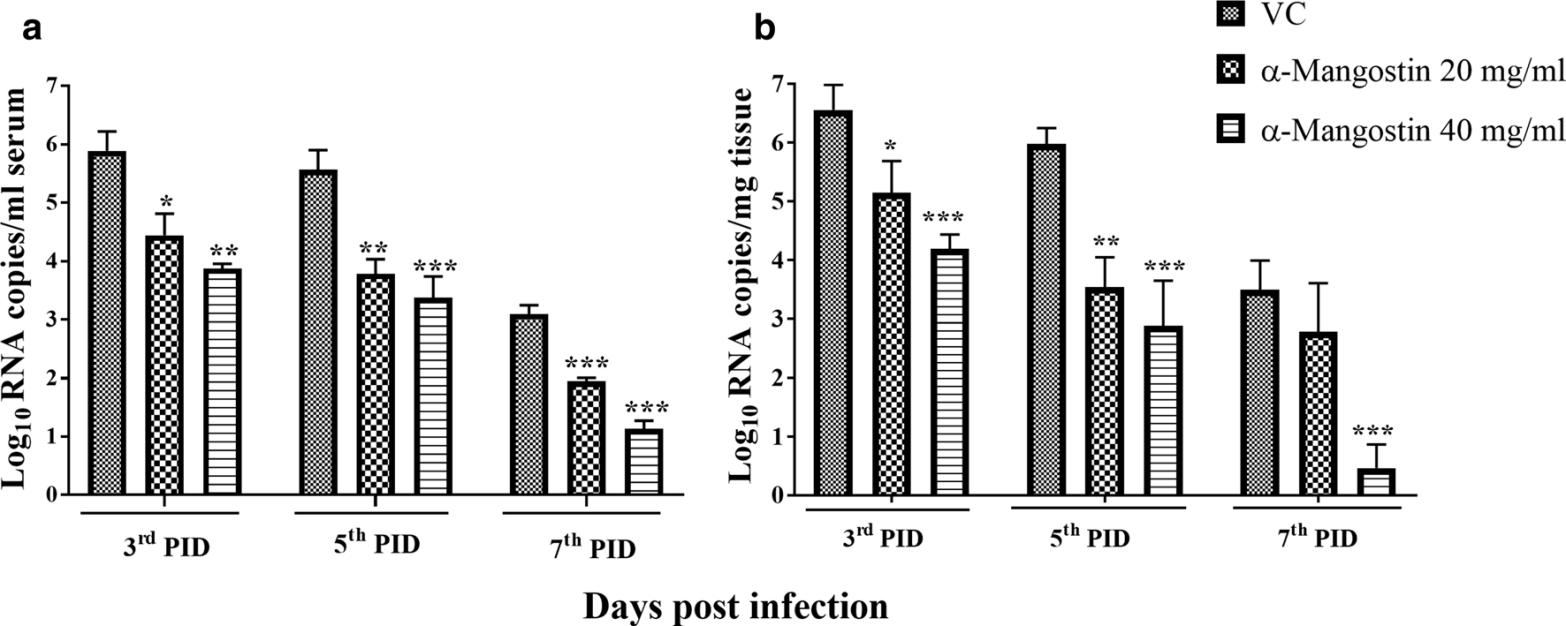
In vivo inhibition of CHIKV using α-Mangostin. **a** The reduction in CHIKV RNA copies/ml in serum and **b** copies/mg muscle tissue of C57/BL6 mice. Mice were infected with CHIKV by intramuscular route and viral RNA copies were assessed in serum and muscles on the 3rd, 5th and 7th day post infection. Two doses of α-Mangostin 20 mg/kg and 40 mg/kg were administered at 1, 2 and 3 dpi via the intraperitoneal route. Values are given as log_10_ RNA copies/ml for serum and copies /mg for muscle tissue. Data shown are mean ± SD. ***p < 0.001; **p < 0.01 and *p < 0.05 vs. vehicle control

α-Mangostin treatment exhibited significant reduction in the serum viral RNA load. At 3rd dpi, α-Mangostin high dose group showed 2.1 log_10_ reduction (99.2% reduction) of viral RNA compared to untreated animals (VC) (*p* < 0.05). At 5th dpi, reduction in viral RNA copy number with the low dose of α-Mangostin was 1.8 log_10_ (*p* < 0.01) and with the high dose of α-Mangostin group was 2.23 log_10_ (99.4% reduction) (*p* < 0.001). At 7th dpi, with the high dose treatment, the reduction in viral RNA was retained at the same level (2.22 log_10_) compared to VC (*p* < 0.001) (Fig. 3a).

The reduction in the CHIKV copies in muscle tissue from α-Mangostin treated mice was observed from day 3 (Fig. 3b). At 3rd dpi, low dose and high dose treatment of α-Mangostin resulted in the reduction of 1.41 log10 and 2.36 log_10_ (99.56% reduction) CHIKV RNA copies respectively in the tissue supernatant. At 5th dpi, 2.44 log10 and 3.1 log_10_ (99.91% reduction) reduction in CHIKV RNA copies was observed in mice treated with low dose and high dose of α-Mangostin. On 7th dpi. no significant reduction in viral RNA was observed in the low dose α- Mangostin treated mice while in high dose α- Mangostin treated group, reduction of 3.4 log_10_ (99.96% reduction) CHIKV RNA copies was observed compared to VC group.

**Fig. 4:**
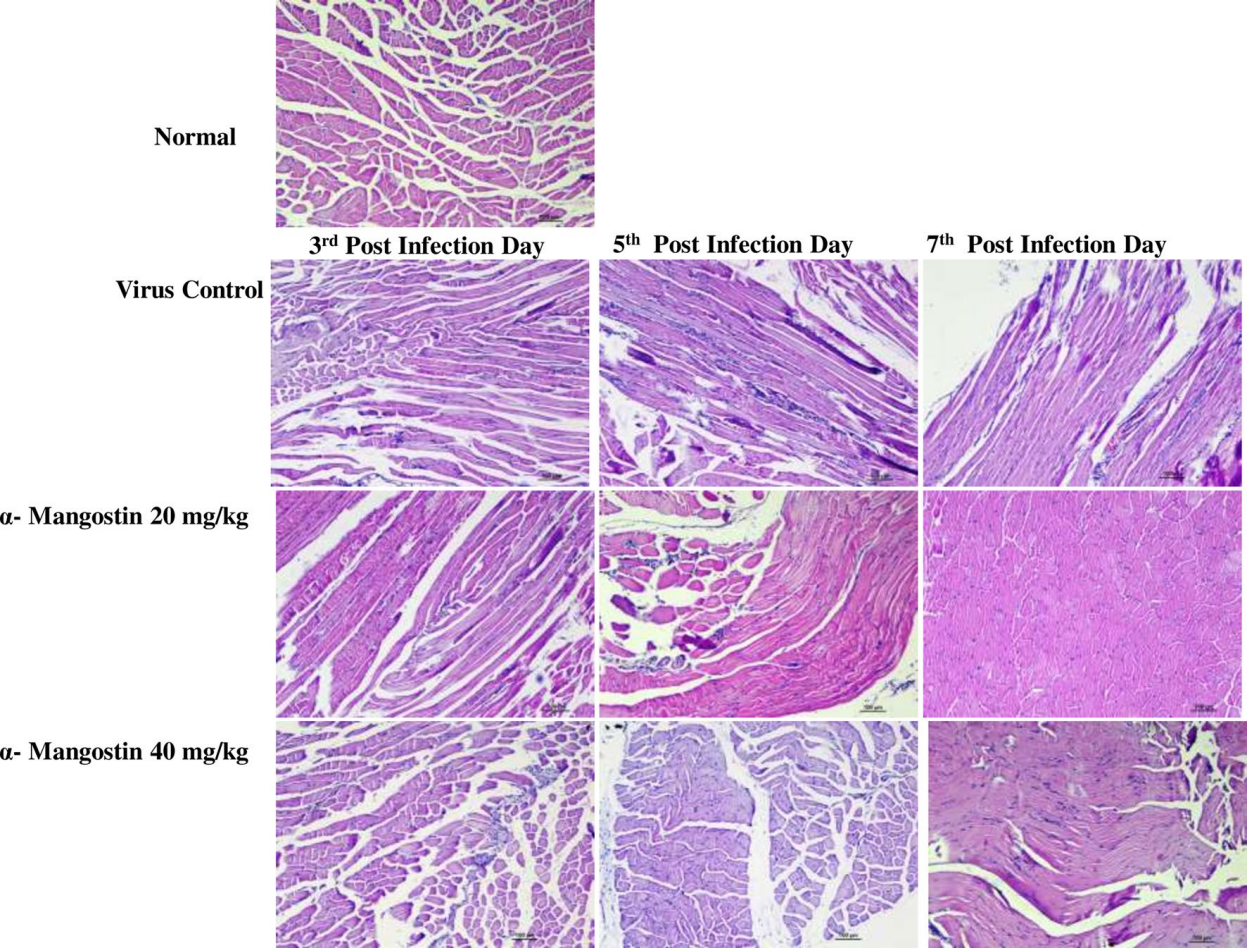
Histopathological changes in mouse muscle tissues after chikungunya infection and α-mangostin treatment. C57BL/6 mice were infected with CHIKV. Hematoxylin and eosin-stained tissue sections were screened to investigate the therapeutic effects of α-mangostin treatment in CHIKV infected mice. PBS injected mice showed normal cellular organization. CHIKV infected muscles showed marked muscle degeneration, atrophy, MNC infiltration and edema (at day 3, 5 and day 7). Treatment with low dose α-Mangostin showed improvement in inflammatory signs in muscle tissue at 7th dpi compared to the VC group. High dose α-Mangostin treated mice muscle tissues showed the regeneration after treatment

Histopathological studies revealed that CHIKV infected muscles showed marked muscle degeneration, atrophy, MNC infiltration and edema (at day 3, 5 and day 7) compared to muscle tissues of normal mice. Compared to the control (normal or untreated mice muscle tissue), presence of mononuclear inflammatory cellular infiltration in between the muscle bundles and coagulative and degenerative changes in the muscle fiber (with loss of continuity in length) are clearly observable in magnified close-ups while these signs are improved in the dose of 40 mg/kg treated α-mangostin group. Treatment with low dose α-Mangostin showed improvement in inflammatory signs in muscle tissue at 7th dpi compared to VC group. High dose α-Mangostin treated mice muscle tissues showed the regeneration after treatment (from 5 dpi onwards) (Magnification X100) (Fig. 4).

### Interaction of α-Mangostin with CHIKV protein targets based on docking studies

**Fig. 5:**
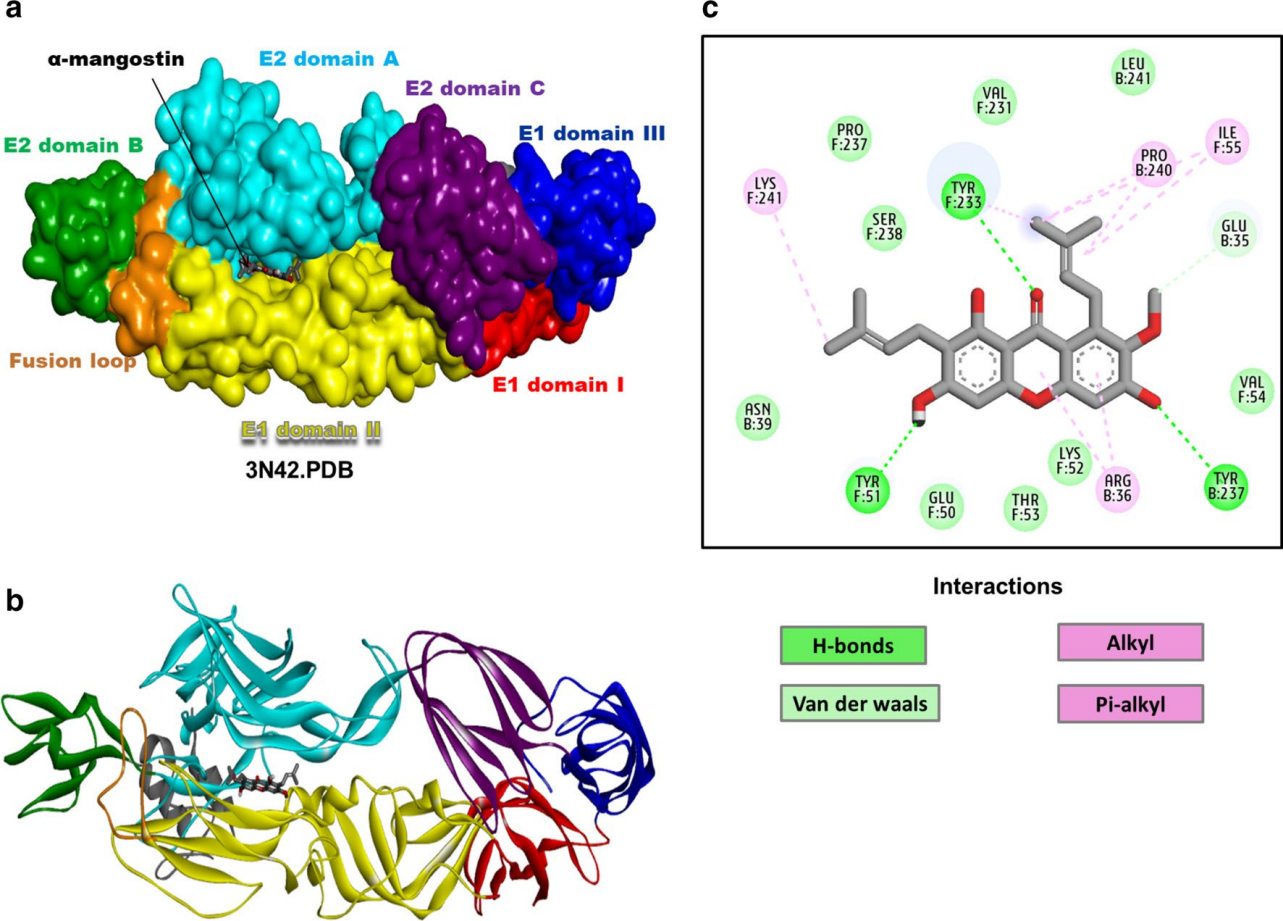
Molecular interactions of α-Mangostin with CHIKV mature envelope glycoprotein complex (3N42.pdb). Interaction of mature envelope glycoprotein complex with α-Mangostin depicted using **a** solvent surface rendered view (probe radius 1.8 Å), **b** solid ribbon form (different colours in both surface and ribbon view represent the different domains of E1, E2 and E3 protein, α-Mangostin represented as stick model coloured by element, **c** 2-dimensional interaction diagram. Different types of interactions are represented by different colours mentioned in the interactions colour panel. All interactions are visualised and analysed using Biovia Discovery studio client 2017

Results based on the docking of α-Mangostin with the mature envelope complex revealed that α-Mangostin docked to a potential binding site between the E1 domain II and E2 domain A that connects to the E2 ß-ribbons. This binding site was surrounded with amino acid residues from E1: Ala33-Arg38 and Gln236-Arg244. The ligand could bind to the cavity with binding affinity (− 8.6 kcal/mol) and stabilize the complex with strong non-covalent molecular interactions. Hydrogen bond interactions were noted with E1: Tyr233, Tyr51 and E2: Tyr237. While pi-alkyl interactions were observed between E2: Arg36 and the cyclic ring of the ligand. Alkyl interactions were formed between E2: Pro240, E1: Ile55,Lys 241 and the prenyl group of α-Mangostin (Fig. 5a–c).

**Fig. 6:**
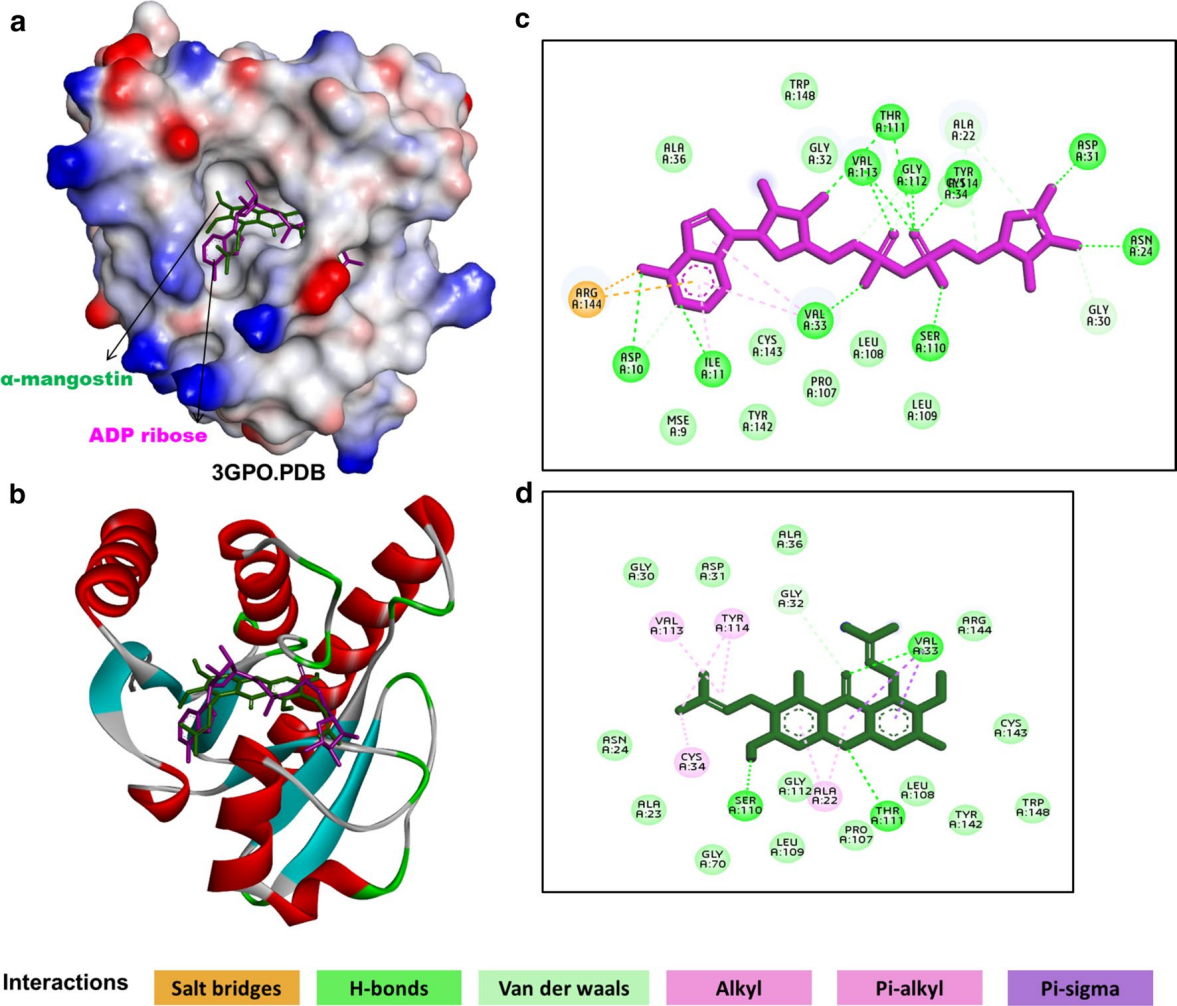
Molecular interactions of α-Mangostin with CHIKV nsP3 macrodomain (3GPO.pdb). **a** Solvent surface view (colored by interpolated charge with a probe radius of 1.8 Å), **b** ribbon view depicting superimposition of re-docked pose of ADP-ribose (represented in a pink stick model) and docked pose of α-Mangostin (green stick model) in the nsP3 macrodomain cavity, **c** 2-dimensional interaction diagram of co-crystallized ADP-ribose with nsP3 macrodomain, **d** 2-dimensional interaction diagram of α-Mangostin with nsP3 macrodomain. Different types of interactions are represented by different colours mentioned in interactions colour panel. All interactions are visualised and analysed using Biovia Discovery studio client 2017

The docking analysis of CHIKV nsP3 macrodomain with α-Mangostin and its natural substrate ADP-ribose showed that both the ligands bind to the same binding site with almost equivalent binding affinity of − 9.3 kcal/mol and − 9.4 kcal/mol, respectively (Fig. 6a). However, it was noted that α-Mangostin binds with comparatively stronger intermolecular interactions than ADP-ribose. In the α-Mangostin bound receptor complex, among eight interacting residues, five participated in pi-alkyl and alkyl interactions. The strongest pi-sigma interaction was observed between Val33 and the central as well as adjacent aromatic ring of α-Mangostin. The other residues involved were Ala22, Cys34, Val113, Tyr114, Arg144 which were observed to form hydrogen bonds (Fig. 6b, c).

Docking of CHIKV peptidase C9 domain of nsP2 protein with α-Mangostin, showed weaker binding interactions (binding affinity − 7.1 kcal/mol) as it completely lacked interaction with the catalytic dyad residues Cys 1013 and His1083 and only one hydrogen bonding interaction was observed with Trp1084 which was in the immediate vicinity to the catalytic residue (Additional file 2: Fig. S2a and S2b). The docking interaction of α-Mangostin with the other CHIKV modelled targets, nsp2 RNA helicase enzyme domains, nsp4 RdRp domain and nsp1 methyltransferase showed binding affinities − 6.9 kcal/mol, − 7.7 kcal/mol and − 7.8 kcal/mol respectively (Additional file 3: Fig. S3). Only one hydrogen bond was noted with Asn30 in case of nsP2 helicase and two hydrogen bonds were observed with Glu64 and Tyr224 in case of nsP1 (Additional file 3: Fig. S3a and Fig. S3c). The docking analysis of CHIKV nsp4 RdRp domain with α-Mangostin showed that it forms three hydrogen bonds with residues Gln392, Arg396 and Met198 (Additional file 3: Fig. S3b).

## Discussion

As a potential antiviral compound, Alpha-Mangostin interferes with HIV-1 virus replication cycle by inhbiting the protease activity [24]. α-Mangostin has also been shown to inhibit DENV in hepatocellular carcinoma cells. The increasing incidences of CHIKV and DENV co-infections [35] indicated that it was worth testing the efficacy of α-Mangostin against CHIKV.

In the current study, α-Mangostin displayed a dose-dependent inhibition of CHIKV replication and considered as having the potential for use as a therapeutic agent against CHIKV infection. Based on the virus yield in terms of viral RNA or FFU, it could be seen that 8 µM α-Mangostin inhibited CHIKV efficiently when added before infection or together with the virus. The reduction in viral RNA expression compared to infectious virus titre is less since the real time RT-PCR is more sensitive than FFU assay and detects RNA from noninfectious virus particles also [36]. Postinfection addition of α-Mangostin also showed moderately reduced anti-CHIKV activity. On the other hand, for DENV infection, α-Mangostin showed posttreatment effectiveness at a concentration of 15–20 µM, though the prophylactic effectiveness was not investigated [28]. The in vitro findings suggest that α-Mangostin may be effective during the early stages of CHIKV infection indicating that α-Mangostin might impair cell receptor mediated endocytosis of the virus. This study for the first time clearly shows the efficacy of α-Mangostin in inhibiting CHIKV replication in mice infected with the CHIKV. The purity of the compound used in the study though not quantified (weight/weight), is high for a natural compound. However, as the antiviral activity could be confirmed by different batches of the compound, it may be assumed that the impurities might not have contributed to the antiviral activity.

To investigate the efficacy of α-Mangostin treatment against CHIKV infections in vivo*,* we employed the previously established intramuscular route for virus infection in the C57BL/6 mice [28]. Replication of CHIKV in skeletal muscle cells is a critical mediator of CHIKV pathogenesis. Published evidence has established skeletal muscle as an important site in CHIKV disease progression [37, 38]. Hence in this study beside serum, muscle tissue were also used for analyzing CHIKV titer. *In-vivo* study using C57BL/6 mice demonstrated that treatment of CHIKV infected mice with α-Mangostin results in effective reduction of viral RNA in serum and muscle tissues.

α-Mangostin showed both time and dose dependent reduction in viral load. Our findings were consistent with the histopatholgical analysis of skeleton muscle tissue of mice. Histopatholgy analysis revealed that α-Mangostin treatment reduced inflammatory infiltration, inflammation and muscle tissue necrosis which corresponded to reduced viremia and viral antigen expression in infected tissues. These results showing reduced viral RNA in serum and muscles, inflammation and tissue damage, indicate the therapeutic effect of α-Mangostin in CHIKV-infected mice. The effect of α-Mangostin in CHIKV infected mice is in contrast to the effects observed in vitro where inhibitory effect was more prominent in pre and cotreatment conditions. This observation suggests that α-Mangostin apart from interacting with the virus might also contribute to modulation of the immune response which needs further investigation [39]. Since α-Mangostin is known to inhibit nuclear factor kappa B (NF-κB) activation, it can also be speculated, that the drug blocks the viral requirement for NF-κB during replication [40]. However, further studies would be needed to confirm this possible mode of inhibition.

The entry of the virus into the cell mainly includes the pH-dependent endosomal membrane fusion between the CHIKV envelope complex and the host cell [41]. The functional analysis of the envelope glycoprotein complex revealed a potential binding site of extreme significance between E1 domain II and E2 domain A in both the mature and the immature forms (data not shown) of the envelope glycoprotein [42–44]. In the former case, upon exposure to low pH in the endosome, this region becomes disordered which facilitates exposure of the fusion loop by removal of the B cap within the E2 domain, resulting in dissociation of the E2–E1 heterodimer [43]. Thus, α-Mangostin when binding to this site may stabilize the acid dependent destabilization of E2–E1 heterodimer and vitiate the fusion process. In the immature envelope complex the potential binding site might act as an indirect allosteric site for inhibition of cleavage of the furin loop [44]. The ligand binding to this site may impair the furin-susceptible envelope protein maturation step as well as block the access of crucial residues required for receptor binding and subsequent inhibition of membrane fusion process. The docking results thus suggests that α-Mangostin may be effective during the early stages of CHIKV infection by impairing cell receptor mediated endocytosis of the virus and can explain the in vitro findings of CHIKV inhibition, under pre-treatment and co-treatment conditions. The experimental substantiation of early phase inhibitory effect of α-Mangostin is an added advantage to this study over earlier studies showing inhibitory potential of screened database compounds based on purely an in silico method. Further experimental validation of the envelope glycoprotein as a potential target needs to be undertaken using the target-specific assay.

The possible interaction between α-mangostin and the possible target of the viral replicase machinery consisting of the non structural proteins was also investigated. The results from docking interaction studies showed that α-Mangostin bound to the same active site where the natural substrate ADP-ribose binds in nsp3 macrodomain. Thus, it can bind competitively to the macrodomain cavity at its substrate ADP-ribose binding site and result in possible impairment of CHIKV replicase activity. Though the binding affinity is noted to be higher when compared to that with the envelope glycoprotein complex, α-Mangostin lacks contact with the crucial residue (Asp10) which is known to play an important role in enzyme activity [10] due to reduced occupancy of the compound in the binding site. It was also observed that another crucial residue, Arg144, interacted with α-Mangostin through weak hydrophobic bonding compared to stronger electrostatic interaction of the same residue in case of nsp3-ADP ribose. This probably explains the reason why α-Mangostin may not completely inhibit the macrodomain activity and showed moderate effect in post-treatment in vitro. However this in silico finding needs to be corroborated by experimental evidence involving replicase-specific assays. Furthermore, docking analysis with nsP2 C9 peptidase domain revealed that α-Mangostin showed poor occupancy in the elongated peptidase cavity of nsP2 domain that facilitates polyprotein binding. Subsequently α-Mangostin was not able to interact with the residues of the catalytic dyad, Cys1013, His1083 crucial for the protease activity of the enzyme [45]. Hence, it is unlikely that α-Mangostin can inhibit protease activity. The RdRp is one of the highly conserved proteins in RNA viruses and the structural motifs such as 315-GDD-317 and the metal binding catalytic site within it facilitates the binding of incoming NTPs for RNA replication and elongation. However α-Mangostin not being a nucleoside/tide analogue does not interact at the conserved catalytic site of the RdRp protein. instead it bound to an allosteric site in the thumb domain of the RdRp structure similar in topology to hepatitis C virus ns5B identified in a previous study [46]. The docking interaction studies of α-Mangostin with nsP2 helicase and nsP1 V-methyltransferase domains indicated that the interacting residues lie within the nsP1-nsP2 interface region [47]. The significance of these observations however needs further investigation, considering that the results are not based on crytal structures for nsp1 v-methyltranseferase domain, nsp2 helicase domain and nsp4 RdRp domain.

## Conclusions

In summary, this study for the first time reports anti-CHIKV potential of α-Mangostin in an in vivo system. The in vitro findings indicate that α-Mangostin can inhibit CHIKV, under pre and cotreatment conditions. In silico studies suggested that α-Mangostin can efficiently interact with multiple CHIKV proteins during different stages of the virus life cycle. Our findings clearly demonstrated that α-Mangostin treatment in CHIKV infected mice reduces viral burden and might help to alleviate disease symptoms in CHIKV-infected mice. Proof of the mechanism of action through likely interactions with the CHIKV mature envelope protein complex and nsP3 macrodomains need to be corroborated by measuring virus internalisation, vesicle acidification or through nsp3-specific functional assays.

Overall the findings from the present study may form the basis to support clinical trials to investigate the possibility of using α-Mangostin to treat chikungunya fever in patients.

## Supplementary Information

**Additional file 1: Figure S1**. Cytotoxicity of α-Mangostin against Vero E6 cells. MTT assay was used to evaluate the cytotoxicity of the compounds. Vero E6 cells were cultured with different concentrations of α-Mangostin for 24 hours. After incubation, MTT solution was added and incubated in the dark at 37°C for 3 h with 5% CO2.. After incubation, the medium was discarded and 100 μL of acidified isopropanol was added to each well and incubated at 37°C for 1 h. The readings were taken in a microplate reader at a wavelength of 570 nm with reference filter at 690 nm. Percentage cytotoxicity or viability was calculated in comparison with cells untreated with α-Mangostin. All experiments were conducted in triplicates.

**Additional file 2: Figure S2**. Molecular interaction of α-Mangostin with nsP2 peptidase C-9 domain (3TRK.pdb) (a) Solvent surface view (probe radius 1.8Å) of docked pose of α-Mangostin (in blue stick model) with nsP2 peptidase C-9 domain (b) 2D interaction diagram of α-Mangostin with nsP2 peptidase C-9 domain showing different intermolecular interactions. All the interactions are visualised and analysed using Biovia Discovery studio client 2017.

**Additional file 3: Figure S3**. Molecular interaction of α-Mangostin with modeled CHIKV targets. The solvent surface rendered view (probe radius 1.6Å) and 2-dimensional interaction diagram showing α-Mangostin (in blue stick model) interacting with (a) nsP2 helicase domain (b) nsP4 RdRP domain and (c) nsP1 methyltransferase domain. All the interactions are visualised and analysed using Biovia Discovery studio client 2017.

## Data Availability

All data generated or analyzed during this study are included in this published article and its supplementary information files.

## Abbreviations

CHIKV: Chikungunya virus
FFU: Foci forming unit
IFA: Immune-fluorescence assay
CHIKF: Chikungunya fever
ZBD: Zinc-binding domain
RdRp: RNAdependent RNApolymerase
GMP: *Garcinia mangostana* Pericarp
IAEC: Institutional Animal Ethics Committee
CPCSEA: Committee for the Purpose of Control and Supervision of Experiments on Animals
DMSO: Dimethyl sulfoxide
qRT-PCR: Quantitative reverse transcription polymerase chain reaction
CMC: Carboxy methyl cellulose
VC: Vehicle control
