# Supplementary figures and images for: In vitro and in vivo studies reveal α-Mangostin, a xanthonoid from Garcinia mangostana, as a promising natural antiviral compound against chikungunya virus
Source: Virol J. 2021 Feb 28;18:47. doi: 10.1186/s12985-021-01517-z (PMC7916311; doi:10.1186/s12985-021-01517-z)

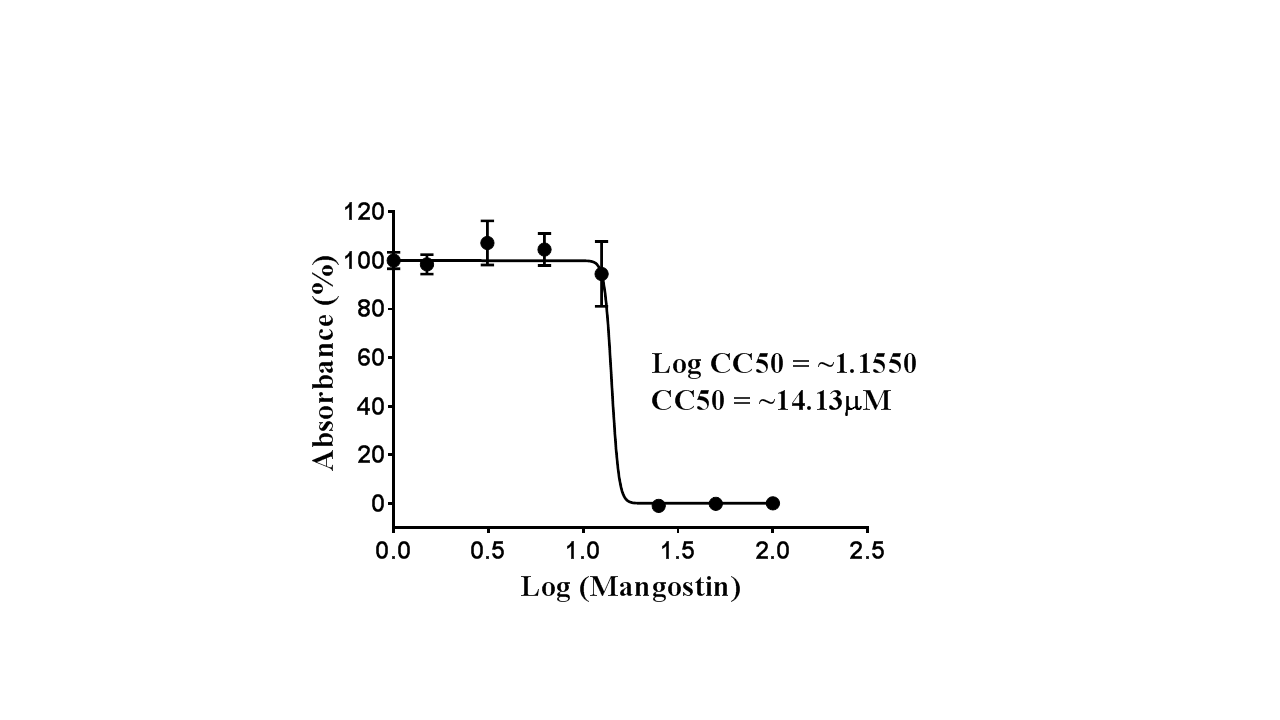

Supplement: Supplementary file 1 — Additional file 1: Figure S1. Cytotoxicity of α-Mangostin against Vero E6 cells. MTT assay was used to evaluate the cytotoxicity of the compounds. Vero E6 cells were cultured with different concentrations of α-Mangostin for 24 hours. After incubation, MTT solution was added and incubated in the dark at 37°C for 3 h with 5% CO2.. After incubation, the medium was discarded and 100 μL of acidified isopropanol was added to each well and incubated at 37°C for 1 h. The readings were taken in a microplate reader at a wavelength of 570 nm with reference filter at 690 nm. Percentage cytotoxicity or viability was calculated in comparison with cells untreated with α-Mangostin. All experiments were conducted in triplicates. [file 12985_2021_1517_MOESM1_ESM.tif]

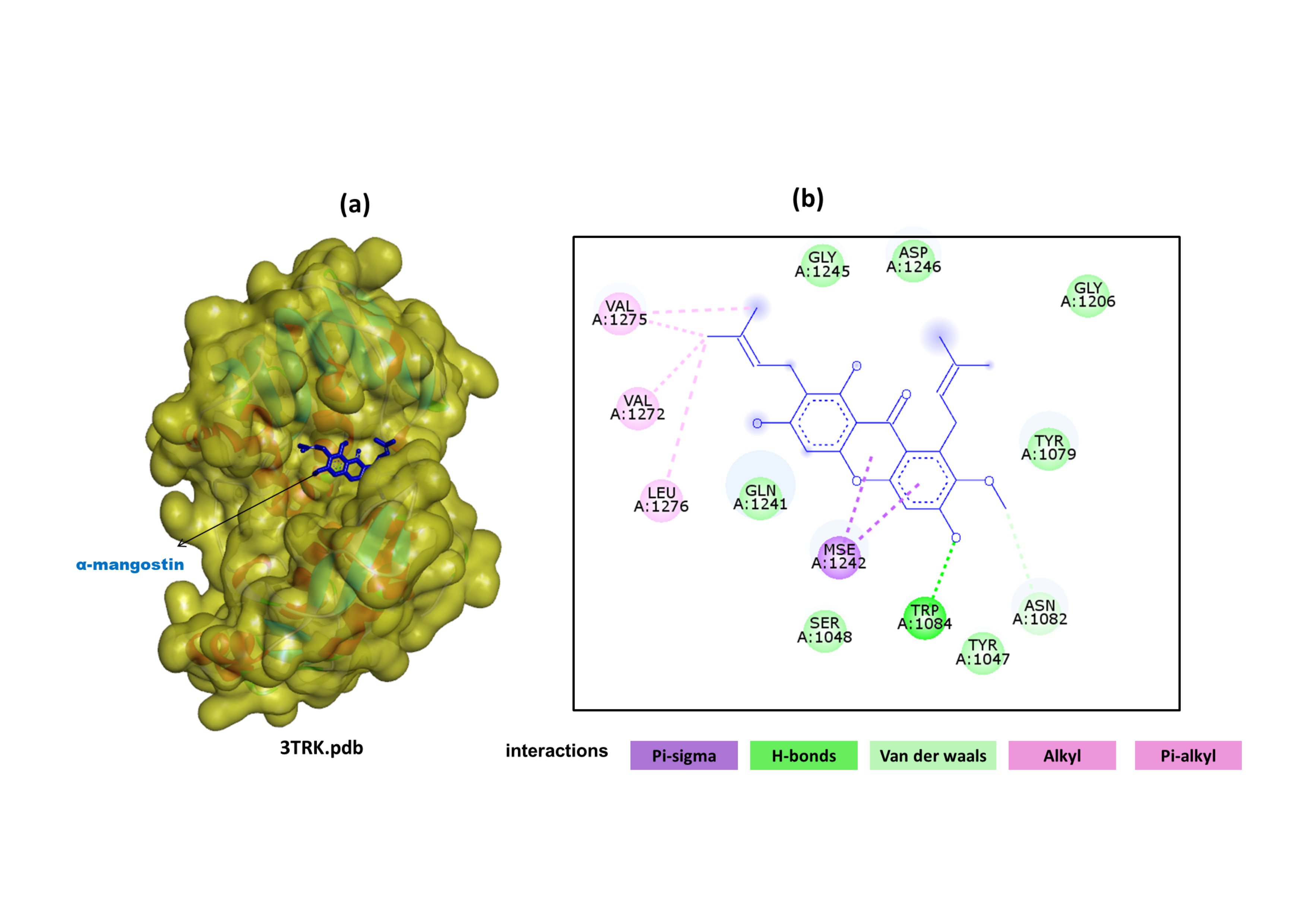

Supplement: Supplementary file 2 — Additional file 2: Figure S2. Molecular interaction of α-Mangostin with nsP2 peptidase C-9 domain (3TRK.pdb) (a) Solvent surface view (probe radius 1.8Å) of docked pose of α-Mangostin (in blue stick model) with nsP2 peptidase C-9 domain (b) 2D interaction diagram of α-Mangostin with nsP2 peptidase C-9 domain showing different intermolecular interactions. All the interactions are visualised and analysed using Biovia Discovery studio client 2017. [file 12985_2021_1517_MOESM2_ESM.tif]

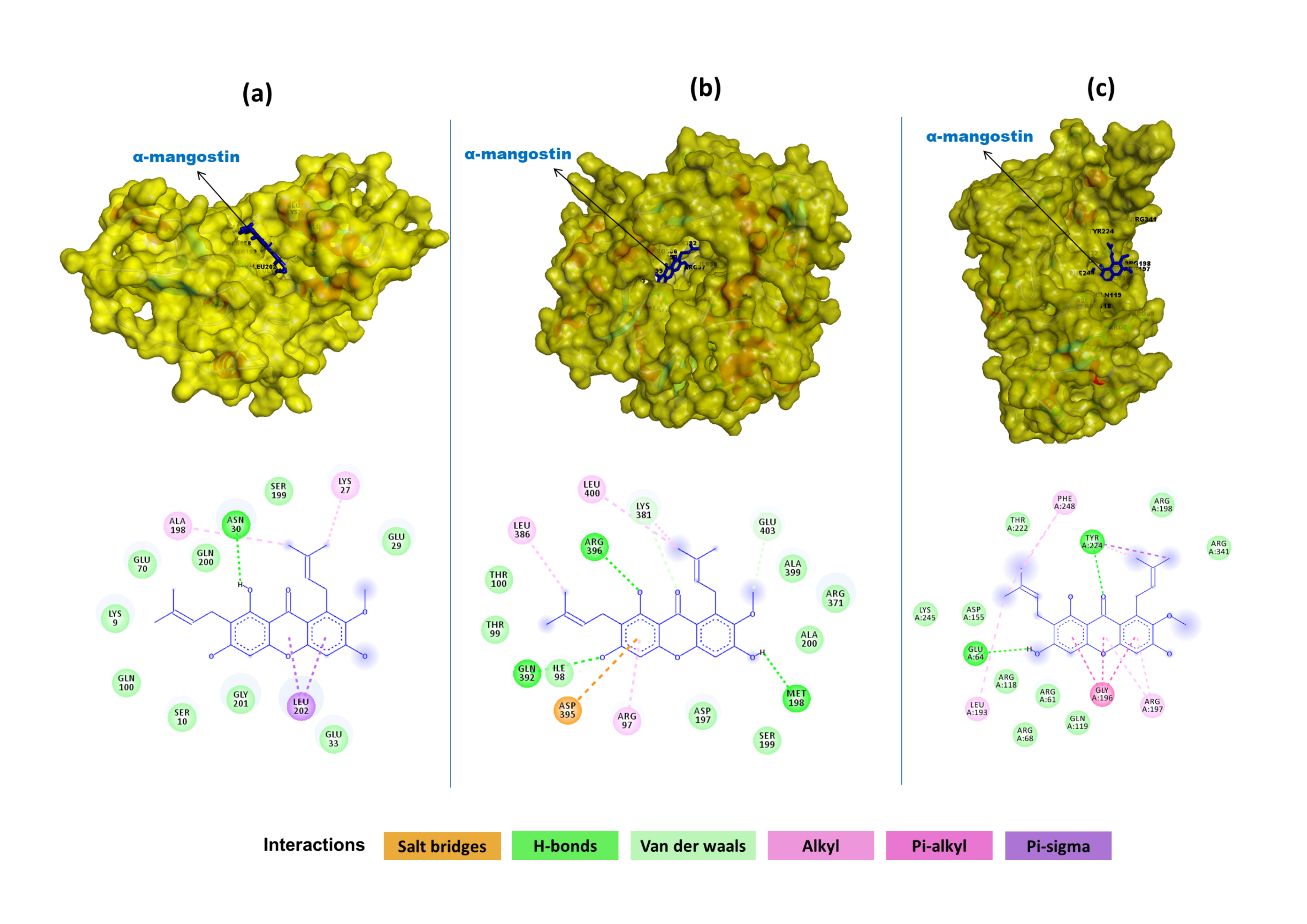

Supplement: Supplementary file 3 — Additional file 3: Figure S3. Molecular interaction of α-Mangostin with modeled CHIKV targets. The solvent surface rendered view (probe radius 1.6Å) and 2-dimensional interaction diagram showing α-Mangostin (in blue stick model) interacting with (a) nsP2 helicase domain (b) nsP4 RdRP domain and (c) nsP1 methyltransferase domain. All the interactions are visualised and analysed using Biovia Discovery studio client 2017. [file 12985_2021_1517_MOESM3_ESM.tif]
